# Inferring polyploid phylogenies from multiply-labeled gene trees

**DOI:** 10.1186/1471-2148-9-216

**Published:** 2009-08-28

**Authors:** Martin Lott, Andreas Spillner, Katharina T Huber, Anna Petri, Bengt Oxelman, Vincent Moulton

**Affiliations:** 1 School of Computing Sciences, University of East Anglia, Norwich, UK; 2 Department of Plant and Environmental Sciences, University of Gothenburg, Gothenburg, Sweden

## Abstract

**Background:**

Gene trees that arise in the context of reconstructing the evolutionary history of polyploid species are often multiply-labeled, that is, the same leaf label can occur several times in a single tree. This property considerably complicates the task of forming a consensus of a collection of such trees compared to usual phylogenetic trees.

**Results:**

We present a method for computing a consensus tree of multiply-labeled trees. As with the well-known greedy consensus tree approach for phylogenetic trees, our method first breaks the given collection of gene trees into a set of clusters. It then aims to insert these clusters one at a time into a tree, starting with the clusters that are supported by most of the gene trees. As the problem to decide whether a cluster can be inserted into a multiply-labeled tree is computationally hard, we have developed a heuristic method for solving this problem.

**Conclusion:**

We illustrate the applicability of our method using two collections of trees for plants of the genus *Silene*, that involve several allopolyploids at different levels.

## Background

Polyploidy is an important evolutionary process in plants, as well as in some animal groups (e.g. [1,2]), accounting for a significant proportion of speciation events [2]. Most eukaryotes have a life cycle which includes a haploid (one set of chromosomes) and a diploid (two sets of chromosomes) part. A *polyploid *can arise from a sterile hybrid which has resulted from the fusion of two incompatible haploid gametes. If, for example due to meiotic errors, the hybrid doubles its chromosomes, it can develop into a new, fertile lineage that is instantaneously reproductively isolated from its parents (but see e.g. [3]), so called *allopolyploidy*. Genome doubling within a lineage is called *autopolyploidy*.

Despite the importance of polyploidy, molecular phylogenetic studies of plants, even at shallow levels where reticulate patterns due to allopolyploidy are to be expected, have been dominated by the use of sequence regions that are unable to trace biparentally inherited evolutionary history. For example, sequences from the cytoplasmatic genomes are usually maternally inherited only, and for nuclear ribosomal DNA it is thought that concerted evolution can eradicate evidence for hybridization (e.g. [4]). Moreover, most phylogenetic studies aiming at tracing polyploid histories use a single nuclear low-copy number gene tree for inference [5-9], or are restricted to relatively simple problems as the origin of allotetraploidy [3,10]. However, to successfully distinguish polyploidization from other biological processes that may be responsible for incongruent phylogenetic patterns (e.g. homoploid hybridization, horizontal gene transfer, incomplete lineage sorting, gene duplication/loss, recombination, sampling or phylogenetic errors), it is desirable to use a large number of gene loci (e.g. [11]). Following this approach, in [12,13] multiple biparentally inherited genes are used for problems involving ploidy levels higher than 4x (tetraploidy) [12,13]. The collections of gene trees arising in such studies commonly have the property that the same species name can label more than one leaf in a single gene tree, due to polyploidization events [5,8-10,12-15]. More formally, we call such trees *multiply-labeled trees *or *MUL-trees*, for short (cf. [16]).

Recently, MUL-trees have been used to construct phylogenetic networks representing the evolutionary history of polyploid species [9]. Although there is now a well-developed algorithm for constructing these networks from a MUL-tree [17], construction of the MUL-tree has to date been performed using an *ad hoc *consensus approach [12], where, essentially, from the given collection of gene trees, a MUL-tree was intuitively constructed in such a way that the number of gene trees that supported each branch was as large as possible. Here we describe a method that generalizes this *ad hoc *approach, and allows the systematic construction of a consensus MUL-tree(s) from a collection of MUL-trees. This method generalizes the greedy consensus method for finding the consensus of a collection of phylogenetic trees [18] although, as we shall see, various complications arise due to computational issues concerning MUL-trees. We illustrate the applicability of our new method using two collections of MUL-trees of flowering plants of the genus *Silene*. An implementation of the method in Java (version 1.5), which is incorporated within the PADRE software package [19], is freely available for download from .

## Results

### The main algorithm

**Figure 1:**
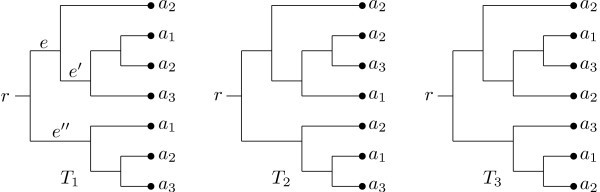
**A collection of MUL-trees**. A collection of three MUL-trees, whose leaves are labeled with the elements of the multiset ℳ = {*a*_1_, *a*_1_, *a*_2_, *a*_2_, *a*_2_, *a*_3_, *a*_3_}. The root of each tree is marked by *r *and in tree *T*_1_, *e *labels a branch (see text for details).

The input to our algorithm consists of a collection of rooted MUL-trees, where the labels that occur are the same for each tree. An example of a collection of three such trees is presented in Figure 1. The leaves of every tree in this example are labeled by *a*_1_, *a*_2_, or *a*_3_. Labels *a*_1 _and *a*_3 _each occur twice, whereas label *a*_2 _occurs three times. To take into account that labels may occur more than once, the leaves of the trees are thus labeled by a *multiset *ℳ, in which the number of occurrences of any label *a *in ℳ is called the *multiplicity *of *a*. For example, in Figure 1 the multiset ℳ is given by {*a*_1_, *a*_1_, *a*_2_, *a*_2_, *a*_2_, *a*_3_, *a*_3_} and the multiplicity of *a*_1 _is 2. We will call a MUL-tree with leaves labeled by a multiset ℳ a *MUL-tree on *ℳ, for short. Note that if all of the labels in ℳ have multiplicity 1, then ℳ is just a set and a MUL-tree on ℳ is a phylogenetic tree in the usual sense [20].

The basic approach taken by our algorithm is to break the input MUL-trees into a collection of *clusters*, that is, sub-multisets of ℳ. In a MUL-tree *T *on ℳ each cluster arises from some branch *e *in *T*, and contains the labels *a *in ℳ with the property that we have to traverse branch *e *on the path from the root *r *to *a *in *T*. We also say that *T exhibits *these clusters. For example, in Figure 1 branch *e *in tree *T*_1 _gives rise to the cluster {*a*_1_, *a*_2_, *a*_2_, *a*_3_}. We then select clusters from those obtained by breaking up the MUL-trees, one at a time, starting with those that are exhibited by most of the input trees, to construct a consensus MUL-tree. At any time the clusters selected by our algorithm so far are chosen to have the property that there exists a MUL-tree that exhibits all of them simultaneously.

**Figure 2:**
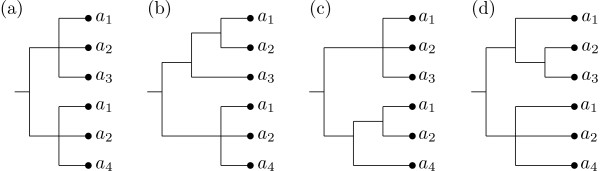
**Adding clusters**. (a) A MUL-tree *T *used to illustrate the difficulties that can occur when adding a cluster. (b)-(c) The cluster {*a*_1_, *a*_2_} can be added in two different ways to the MUL-tree *T*. (d) The cluster {*a*_2_, *a*_3_} can be added to the MUL-tree in (a), but in a unique way.

Note that this approach is also used in the greedy consensus method for constructing a consensus of a collection of phylogenetic trees [18]. In this method the efficient selection of the next cluster to be inserted into the consensus tree is based on the following useful property of phylogenetic trees [21]: If every pair of clusters in a collection is *compatible*, that is, there exists a phylogenetic tree that exhibits both clusters, then there is a (necessarily unique) phylogenetic tree that exhibits the whole collection. In contrast, for MUL-trees this property does not hold in general. For example, among the clusters obtained from the MUL-trees in Figure 1 every pair can be exhibited by some input tree. But it can be checked that there is no MUL-tree that exhibits them all at once. In fact, it is NP-hard to decide whether a collection of clusters of a multiset ℳ can be exhibited by some MUL-tree on ℳ [22]. And, even if there exists such a tree, it need not be unique (see e.g. Figure 2(b) and 2(c)).

To cope with these difficulties, our algorithm first greedily adds in those clusters containing at least one label *a *that has multiplicity 1 in ℳ, called *core clusters*. The key property of these clusters is that, if they can be added to a MUL-tree, then this can only be done in a unique way [22]. For example, the cluster {*a*_2_, *a*_3_} can be added in only one way to the MUL-tree in Figure 2(a) resulting in the MUL-tree depicted in Figure 2(d). We call the MUL-tree obtained by adding in core clusters the *backbone tree*. Note that if every element in ℳ has multiplicity 1 then every cluster is a core cluster and our algorithm works precisely like the greedy consensus method for phylogenetic trees [18]. In general, however, there will be clusters that are not core clusters, called *ambiguous clusters*, and therefore, in the second phase, we continue to greedily select these and, if possible, insert them into the backbone tree. This results in one or more MUL-trees, all of which exhibit the same collection of clusters and that contain the backbone tree as a subtree.

Note that as part of the two-phase strategy outlined above we also apply a threshold *t *that determines the minimum number of input trees that must exhibit a cluster in order to be taken into account when forming a consensus MUL-tree. This threshold helps to prevent a core cluster being exhibited by only a small number of input trees blocking the addition of an ambiguous cluster that is exhibited by many input trees later on. The idea of using a threshold is similar to the approach taken by the majority rule consensus method for phylogenetic trees [18].

### A detailed description of the algorithm

**Figure 3:**
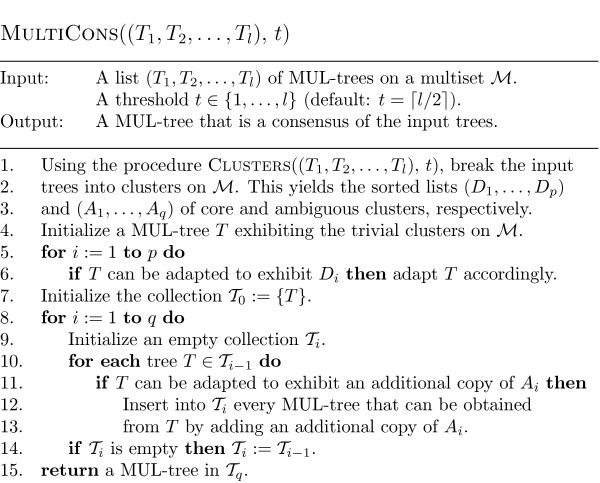
**Pseudo-code for the algorithm MULTICONS**. Pseudo-code for our algorithm that computes a consensus MUL-tree of the input MUL-trees.

**Figure 4:**
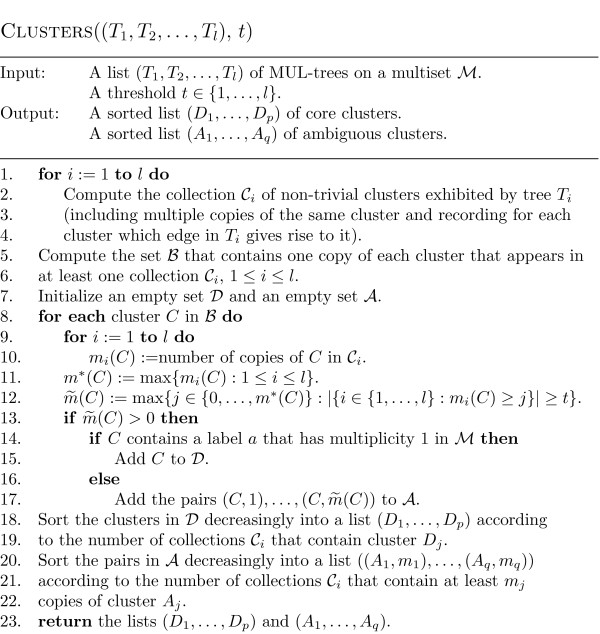
**Pseudo-code for the procedure CLUSTERS**. Pseudo-code for the procedure that computes the sorted list of the non-trivial core and ambiguous clusters, respectively.

We now give a full description of our new algorithm: In Figure 3, we present it in form of pseudo-code. First it uses the procedure CLUSTERS (presented in Figure 4 in form of pseudo-code) to compute the sorted lists (*D*_1_, ..., *D*_*p*_) and (*A*_1_, ..., *A*_*q*_) of core and ambiguous clusters (Lines 1-3) on the given multiset ℳ. As mentioned in the previous section, we then select clusters, one at a time, from these lists to form a consensus of the input trees. We start (Line 4) with a tree *T *that exhibits precisely the *trivial *clusters on ℳ, that is, the clusters containing a single label in ℳ (note that these clusters are exhibited by all of the input trees). Then we construct the backbone tree using the list of core clusters (Lines 5-6), and then add ambiguous clusters to the backbone tree (Lines 7-14). The output is a MUL-tree selected from the resulting collection  of MUL-trees. An output tree from this collection can either be selected by the user, or the whole collection of trees can be returned. In the next section we provide a score function to aid with the tree selection process.

To conclude the description of our algorithm we describe the details of the computation of the core and ambiguous clusters as presented in Figure 4. First, for each input MUL-tree *T*_*i*_, 1 ≤ *i *≤ *l*, we compute the collection of non-trivial clusters  that are exhibited by *T*_*i *_(Lines 1-4). If an input MUL-tree exhibits a cluster *C *several times, e.g. tree *T*_1 _in Figure 1 exhibits the cluster {*a*_1_, *a*_2_, *a*_3_} twice, we include in  the corresponding number of copies of *C *and distinguish them by recording the branch in *T*_*i *_that gives rise to them.

Next we combine the collections  into a set ℬ of all clusters that arise from the branches of the input MUL-trees without taking multiple copies of the same cluster into account (Lines 5-6). Then using the threshold *t*, for each cluster *C *in ℬ the number (*C*) is computed, which is the largest number of copies of *C *such that at least *t *of the collections  contain that many copies of *C *(Lines 8-12). The clusters *C *in ℬ with (*C*) > 0 are then partitioned into core clusters and ambiguous clusters (Lines 13-17).

The core clusters are collected together in the set  (Line 15). Note that if *D *is a core cluster then (*D*) ≤ 1 holds. In contrast, for an ambiguous cluster *A *one can have (*A*) > 1, and so we record the numbers of copies of *A *that we might be able to accommodate in the consensus tree in the form of pairs (*A*, 1), ..., (*A*, (*A*)) denoting the resulting set of pairs by  (Line 17). The core clusters *D *in  are then sorted decreasingly according to the number of collections among  in which cluster *D *is contained (Lines 18-19), where ties are broken arbitrarily. This yields a sorted list (*D*_1_, ..., *D*_*p*_) of core clusters. Similarly, the pairs (*A*, *m*) in  are sorted decreasingly according to the number of collections among  that contain at least *m *copies of *A *(Lines 20-22). Again ties are broken arbitrarily. This yields a sorted list ((*A*_1_, *m*_1_), ..., (*A*_*q*_, *m*_*q*_)) of the pairs in  from which the sorted list (*A*_1_, ..., *A*_*q*_) of ambiguous clusters is extracted, with some clusters possibly occurring more than once in this list.

The run time of our algorithm can be bounded in terms of the number *l *of input trees, the sum *m *of the multiplicity of all elements in ℳ, and the sum *d *of the multiplicity of all elements in ℳ except those that occur with multiplicity 1. For example, for the multiset labeling the tree in Figure 2(a) we have *m *= 6, which is the same as the number of leaves of the tree, whereas *d *= 4 because the multiplicity of *a*_3 _and *a*_4 _is not taken into account. Note that, since the number of branches of a tree is linear in the number of its leaves, the total number of clusters (core and ambiguous) is in *O*(*ml*). Hence a straightforward implementation of the procedure CLUSTERS in Figure 4 has a run time in *O*(*m*^2^*l*^2^).

Once the lists of clusters have been computed, the main task is to check, for a given tree and a cluster, whether the tree can be adapted to exhibit the additional cluster. Basically we can use the algorithm presented in [[22], ch. 5] for this task, which yields a run time for MULTICONS of *O*(*m*^2^*l*^2 ^+ *m*^4^*l *+ *d*^*d*^*ml*). The first term in this bound comes from the run time of the procedure CLUSTERS. To give the reader some idea how the remaining two terms in the bound arise, note first that checking whether a core cluster can be added in Line 6 of the algorithm MULTICONS can be done by going through the vertices of degree higher than 3 in the tree constructed so far and checking whether they can be resolved to accommodate the additional cluster. Implementing this in a straightforward way, each core cluster can be checked in *O*(*m*^3^) time and the resulting tree is, as mentioned above, unique. Since there are *O*(*ml*) clusters, this yields the second term.

As for the third term, checking whether an ambiguous cluster can be added to a particular tree in Lines 12-13 can be done in a similar way as outlined above for core clusters. However, the key difference is that there are now *O*(*d*) resulting trees. This implies that the number of trees in the output collection  is bounded by *O*(*d*^*d*^), in view of the fact that the number of non-trivial ambiguous clusters in a MUL-tree on ℳ is in *O*(*d*) (see [22] for more on this). This leads to the last term in the bound above.

### Pre- and postprocessing

It often happens that the collection of input MUL-trees are labeled by slightly different multisets (due e.g. to sequencing difficulties or lack of sampling). Hence, we have also developed a simple preprocessing procedure that essentially restricts the input trees to a common multiset ℳ of labels. This procedure employs a majority rule, that is, for every label *a *that appears in at least one of the input trees, the multiplicity of *a *in ℳ is chosen as the largest integer *m *such that *a *appears in at least half of the input trees with multiplicity at least *m*. Note that it is possible that some labels appear in so few input trees that they have multiplicity 0 in ℳ, that is, they will not appear in the consensus tree.

Once we have determined this multiset ℳ it remains to restrict the input trees to ℳ and to compute a consensus of the restrictions. However, the restriction process might involve a choice of which leaves labeled by copies of a certain label should be removed. As the number of possible choices increases rapidly for different labels, potentially leading to a huge number of different restrictions, we avoid deciding which copies of a label to remove as follows. If a cluster contains more copies of a label than the multiset ℳ, these additional copies are removed from the cluster, independently of all other clusters. Note that this might however yield two copies *C *and *C' *of a cluster in the collection  arising from some input tree *T*_*i *_such that the branch *e *that gives rise to *C *lies on the path from the root of *T*_*i *_to the branch *e' *that gives rise to *C'*. For example, consider the tree *T*_1 _in Figure 1. Branch *e *gives rise to the cluster {*a*_1_, *a*_2_, *a*_2_, *a*_3_} and branch *e' *to the cluster {*a*_1_, *a*_2_, *a*_3_}. However, if we restrict *T*_1 _to the multiset {*a*_1_, *a*_1_, *a*_2_, *a*_3_, *a*_3_}, say, we remove one copy of *a*_2 _from {*a*_1_, *a*_2_, *a*_2_, *a*_3_}, then after the restriction both *e *and *e' *give rise to the same cluster {a_1_, *a*_2_, *a*_3_}. In this situation the cluster arising from *e' *is not counted as an additional copy and can rather be viewed as an artifact of the restriction to ℳ and will, therefore, not be included in . So, for the tree *T*_1 _in Figure 1 only the two copies of cluster {*a*_1_, *a*_2_, *a*_3_} that arise from branches *e *and *e" *will be taken into account.

We also developed a postprocessing procedure that scores the MUL-trees in the collection  computed by the algorithm MULTICONS, to deal with the fact that this collection could be quite large. The basic idea for the scoring is to estimate the number of allopolyploidization events that are implicitly hypothesized by a MUL-tree. To do this, we use an algorithm presented in [17], called MULTIBUILD, to compute for each MUL-tree *T *in  a network (*T*) representing *T*. We then use the number of allopolyploidization events hypothesized by (*T*) to score each MUL-tree *T*, since (*T*) can be viewed as a most parsimonious representation of *T *in terms of such events. In practice, we have found it best to take all possible refinements of *T *to bifurcating trees and to calculate the minimum number of allopolyploidization events in the networks obtained for those refinements as the score of *T*. We suspect that this is because MULTIBUILD is only guaranteed to find an optimal network with respect to the number of allopolyploidization events if the MUL-tree is binary.

### Applications

We illustrate the application of our method, along with some of the complexities involved in constructing a consensus of MUL-trees, using two data sets of MUL-trees of the flowering plant genus *Silene *(Caryophyllaceae). These examples were computed using the implementation of the algorithm in the PADRE package. This takes as input a collection of trees in NEWICK format [23], and displays the resulting consensus tree, which can also be saved as a file in encapsulated postscript (eps) or NEWICK format.

**Figure 5:**
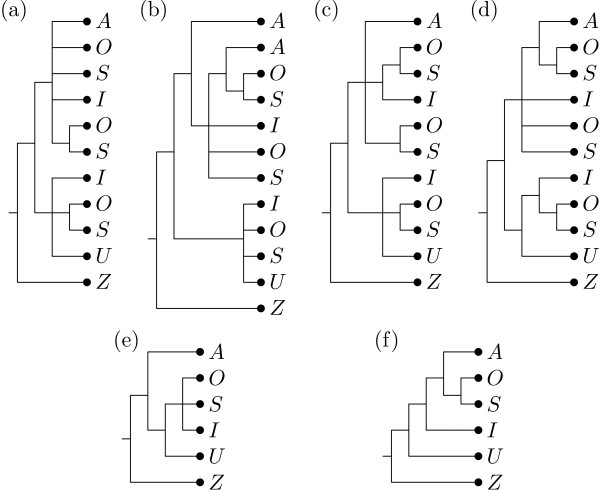
**Input trees for first example**. Six MUL-trees, restrictions of gene trees originally published in [12], that we used as input for our algorithm.

The first collection of MUL-trees we apply our algorithm to is depicted in Figure 5. We chose this example, as it is small enough to easily follow the workings of the algorithm. The labels represent *Silene *species, namely, the diploids *S. ajanensis *(*A*) and *S. uralensis *(*U*), the tetraploid *S. involucrata *(*I*), and the two hexaploids *S. sorensenis *(*S*) and *S. ostenfeldii *(*O*). All trees are rooted at *S. zawadskii *(*Z*). They are restrictions of the larger gene trees published in [12] to the species *A*, *U*, *I*, *S*, *O *and *Z*.

The gene trees in [12] are reconstructed using standard techniques in phylogenetic analysis from regions of the nuclear RNA polymerase (RNAP) gene family (RPB2, RPA2, RPD2a, and RPD2b, Figure 5(a)-(d)), two concatenated chloroplast regions (*rps16 *intron and the *psbE*/*petG *spacer, Figure 5(e)), and one nuclear ribosomal region (ITS1 and ITS2, with the intervening 5.8S gene, Figure 5(f)). Although all 6 gene trees in Figure 5 may be viewed as MUL-trees, it should be noted that only the four trees on the RNAP genes (Figure 5(a)-(d)) are true MUL-trees. For the chloroplast regions, this is because chloroplasts are maternally inherited and harbor a haploid genome. A MUL-tree constructed for such regions is therefore a phylogenetic tree in the usual sense. Regarding the nuclear ribosomal DNA, the reason is different in the sense that, although they constitute a very large multigene family, its members are kept identical or very similar by concerted evolution. Therefore, traces of hybridization events are quickly eradicated (e.g. [4]). As a consequence, nuclear ribosomal DNA can behave similarly to a haploid, uniparental locus.

When we apply our algorithm to the input MUL-trees in Figure 5, we first apply the preprocessing procedure to compute a multiset ℳ to which we restrict the input trees, yielding ℳ = {*A, I, I, O, O, O, S, S, S, U, Z*}. Using this multiset and the default value of ⌈l/2⌉ = 3 for the threshold *t *we obtain the 4 non-trivial core clusters



and the non-trivial ambiguous clusters {*O, S*} (two copies) and {*I, O, S*} (one copy) to build a consensus tree. Note that, although the cluster {*A, I, O, O, S, S*} is generated twice when breaking *T*_2 _into clusters (since it is exhibited by *T*_2 _and also results from restricting the exhibited cluster {*A, A, I, O, O, S, S*} to ℳ by removing one copy of label *A*), it is taken into account only once. Also note that the choice of the threshold *t *implies, for example, that even though {*A, I, O, S*} is a core cluster it is not taken into consideration for constructing the backbone tree as this cluster is only exhibited by a single input tree, namely the tree in Figure 5(f).

**Figure 6:**
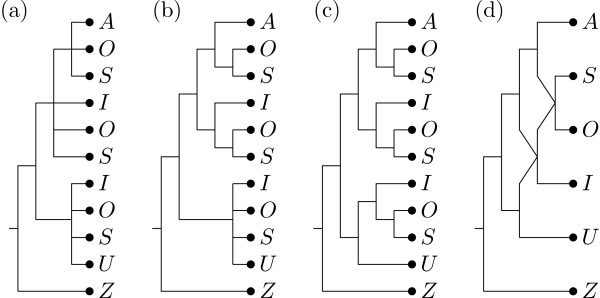
**Output for first example**. (a) Backbone tree using the default value for threshold *t*. (b) One of the three MUL-trees obtained by adding ambiguous clusters to the backbone tree. (c) A possible resolution of the tree in (b) to a bifurcating tree. (d) The reticulate network constructed from the tree in (c).

The backbone tree constructed from the 4 selected core clusters is depicted in Figure 6(a). Adding in the ambiguous clusters results in 3 semiresolved consensus MUL-trees one of which we depict in Figure 6(b). When applying the scoring procedure, by constructing a reticulate network with MULTIBUILD for all 32 distinct refinements of these 3 trees to bifurcating trees, we find a single refined MUL-tree with minimum score which is depicted in Figure 6(c). Note that this tree was also constructed by the *ad hoc *method mentioned above in [12]. The reticulate network computed for this MUL-tree is depicted in Figure 6(d). It postulates 2 consecutive allopolyploidization events, the first one resulting in the tetraploid *S. involucrata *and the second one leading to the two hexaploids *S. sorensenis *and *S. ostenfeldii*.

**Figure 7:**
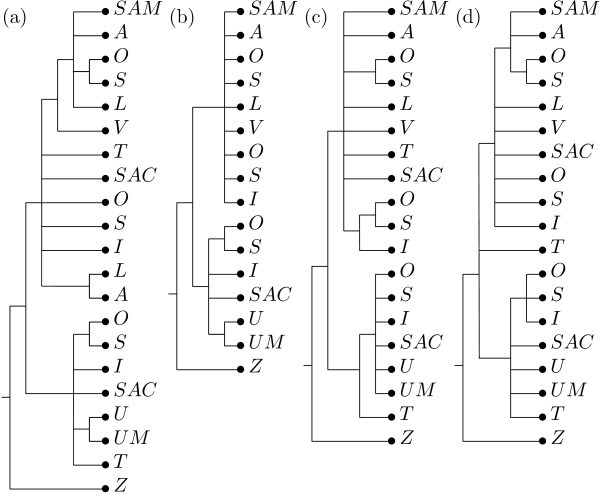
**Input trees for second example**. The second collection of MUL-trees we apply our algorithm to, involving additional *Silene *species.

The second collection of MUL-trees we applied our algorithm to is depicted in Figure 7. This collection is more complex than the previous one since it involves more species and the trees are much more unresolved. The additional *Silene *species appearing in the trees (again represented by their label) are: *S. linnaeana *(*L*), *S. uralensis *(Mongolia) (*U M*), *S. samojedora *(*SAM*), and *S. villosula *(*V*), which are all diploid, and *S. sachalinensis *(*SAC*) and *S. tolmatchevii *(*T*), whose chromosome numbers are unknown but, in view of the number of RNAP gene copies found, are likely to be tetraploids. It should be noted that in contrast to the trees in Figure 5, the four MUL-trees in Figure 7 were reconstructed solely from RNAP gene families (i.e. RPB2 (a), RPA2 (b), RPD2a (c), and RPD2b (d)). As before, all MUL-trees are rooted at *S. zawadskii *(*Z*).

**Figure 8:**
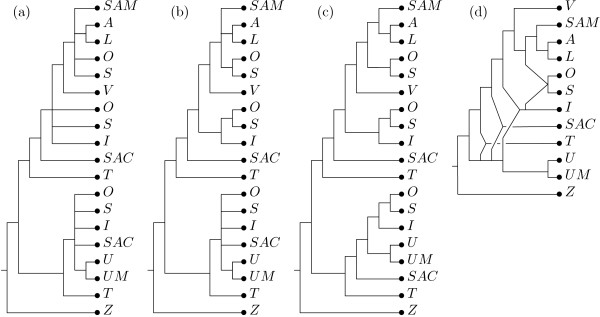
**Output for second example**. (a) Backbone tree using threshold *t *= 1. (b) One of the 6 MUL-trees obtained by adding ambiguous clusters to the backbone tree. (c) A possible resolution of the tree in (b) to a bifurcating tree. (d) The reticulate network constructed from the tree in (c).

The multiset of labels constructed by the preprocessing procedure is ℳ = {*A, I, I, L, O, O, O, S, S, S, SAC, SAC, SAM, T, T, U, U M, V, Z*}. Using this multiset a collection of 15 non-trivial clusters is derived from the input trees, of which 12 are core clusters and 3 are ambiguous clusters. We employed a threshold of *t *= 1, as the input trees are very unresolved and larger thresholds yield only a small number of non-trivial clusters to form a consensus tree. In Figure 8(a) we depict the unique backbone tree constructed from 10 of the non-trivial core clusters. Adding ambiguous clusters to this tree results in 6 semiresolved consensus MUL-trees one of which we depict in Figure 8(b). By exhaustively searching through the set of all 885 refinements of these 6 trees, we find that only 9 of them give rise to a reticulate network with the minimum number of 4 hypothesized allopolyploidization events. In Figure 8(c), we depict one of them and in Figure 8(d) we depict the corresponding reticulate network. Note that this network agrees with the network presented in Figure 6(d) when restricted to the *Silene *species in the first collection. In addition two further allopolyploidization events are hypothesized, suggesting that *S. sachalinensis *and *S. tolmatchevii *are tetraploids.

## Conclusion

In this paper, we have presented a new algorithm for constructing a consensus MUL-tree(s) from a collection of MUL-trees, and illustrated its applicability using two examples. Both consisted of collections of gene trees that were constructed from sequence data of polyploid plants, including biparentally informative sequences. In both cases, we have also obtained networks that provide scenarios for how the plants evolved.

As a preprocessing procedure we provide a way to deal with the situation that some input trees might have missing or additional leaf labels. A key task in this context is to determine the multiset of labels that should appear in the consensus tree. The simplest possible approach would be to just take the union of the multisets over all input trees, that is, every label has the maximum multiplicity with which it occurs in an input tree. However, in practice we found that this tended to lead to an overestimation of the multiplicity of some labels, hence our use of a majority rule procedure. Even so, our approach is still rather simple in that it is only likely to work well in case the number of additional or missing leaf labels is small since otherwise too much information is lost. To circumvent this problem one might try to develop supertree methods for MUL-trees, although we expect that this task would be quite challenging in the light of the fact that many versions of the supertree problem are hard even for collections of phylogenetic trees (see e.g. [24]). In this vein, it might also be of interest to explore the possibility of constructing consensus- or super-networks [25].

The basic idea for our algorithm, that is, breaking the input trees into clusters and then combining some of these clusters to form a consensus tree, seems to yield good results if the input trees are not too unresolved and there are enough clusters that are exhibited by many input trees. However, in some circumstances, the greedy construction involves a random choice of which clusters, exhibited by the same number of input trees, should be added next. In view of this, a more canonical approach to selecting clusters could be desirable. This might be achieved by generalizing, for example, the majority rule consensus approach [18] to MUL-trees. Even so, the results in [22] imply that as the total multiplicity *d *of those labels that appear with multiplicity greater than 1 grows, the majority rule consensus tree will resemble more and more the strict consensus tree, which tends to be very unresolved. Our algorithm tries to address this issue by allowing the user to explore how being strict (large threshold *t*) or generous (small threshold *t*) affects the resulting consensus trees. In addition, there is also the option to explore whether further clusters exhibited by less than *t *input trees could still be added into the consensus tree at the end. In future work, it could also be interesting to try and generalize non-cluster-based approaches for computing a consensus of phylogenetic trees as described in [18] (e.g. by recoding the MUL-trees in some way).

We employ a postprocessing procedure to score the resulting trees, but as this potentially involves refining trees to binary trees, it has a worst case run time that is exponential in the number of leaves of the resulting trees (although the score for a single refined tree can be computed by the algorithm MULTIBUILD in polynomial time). Therefore, despite working quite well for the examples we have considered, it is likely to be limited to rather small problem instances. Moreover, the number of trees with optimal score can be quite high, especially when the input trees are very unresolved. Even though multiple optimal solutions are not uncommon in phylogenetics (e.g. there can be several most parsimonious trees [26]), it could still be of interest to develop ways to systematically select specific optimal trees. For example, alternative score functions could be developed that take into account how the clusters are arranged in the input MUL-trees or, if available, branch length information.

The parameter that seems to have the biggest impact on the run time is the total multiplicity *d *of those labels that appear with multiplicity greater than 1 in many input trees. Even though the theoretical worst case run time of our algorithm increases exponentially with this parameter (which is to be expected due to the inherent computational complexity involved in computing a consensus of MUL-trees), for both examples presented above the run time was only a few seconds on a modern desktop computer.

In view of recent advances in DNA sequencing technologies (e.g. [27]), we anticipate that many more data sets will soon become available giving rise to collections of MUL-trees. The algorithm proposed in this paper will hopefully provide a useful new tool for analyzing such collections.

## Authors' contributions

All authors contributed to the ideas and the development of the algorithm which was implemented by ML. AP and BO provided the biological data set and ensured the biological relevance of the paper. Every author contributed to the writing of the paper.
